# Targeting the interaction between long noncoding RNA XR_001779380 and Prdm1 to enhance IFN-γ immunity in murine neonatal intestinal epithelial cells

**DOI:** 10.1128/mbio.00773-25

**Published:** 2025-06-11

**Authors:** Kehua Jin, Ai-Yu Gong, Shuhong Wang, Gislaine A. Martins, Juliane K. Strauss-Soukup, Roberta M. O'Connor, Xian-Ming Chen

**Affiliations:** 1 Department of Microbial Pathogens and Immunity, Rush University Medical Center 2468, Chicago, Illinois, USA; 2 Department of Biochemistry and Molecular Biology, School of Basic Medical Sciences, Hubei University of Science and Technology 418442, Xianning, Hubei, China; 3 Departments of Medicine and Biomedical Sciences, Research Division of Immunology Cedars-Sinai Medical Center, David Geffen School of Medicine, University of California—Los Angeles 8783, Los Angeles, California, USA; 4 Department of Chemistry and Biochemistry, Creighton University College of Arts and Sciences 357852, Omaha, Nebraska, USA; 5 Veterinary and Biomedical Sciences, College of Veterinary Medicine, University of Minnesota 70195 , St. Paul, Minnesota, USA; University of Wisconsin—Madison, Madison, Wisconsin, USA

**Keywords:** Prdm1, IFN-gamma, antisense oligonucleotides, intestinal epithelium, neonates, LncRNAs, Swi/Snf, Pias1, innate defense, *Cryptosporidium*

## Abstract

Intestinal epithelial cells (IECs) serve as the front line of host defense in the intestine, with IFN signaling playing a critical role in regulating epithelial cell-intrinsic defense against intracellular pathogens. However, IFN-γ-mediated antimicrobial defense is usually reduced in the gastrointestinal tract in infants, and the underlying mechanisms remain unclear. We previously observed that the lncRNA XR_001779380 promotes IFN-γ-stimulated gene transcription in murine IECs. Interestingly, its interaction with Prdm1, a DNA-binding protein expressed in the neonatal but not adult intestinal epithelium, attenuates IFN-γ-stimulated gene transcription, thereby contributing to suppression of IFN-γ-mediated, epithelial cell-intrinsic defense in the neonatal intestine. In this study, we further investigated the role of Prdm1 in suppressing IFN-γ response in murine neonatal IECs. Additionally, we explored the development of specific antisense oligonucleotides to interfere with XR_001779380-Prdm1 interaction to promote IFN-γ response in IECs. Our data show that Prdm1 suppresses IFN-γ-mediated gene transcription, and its induction inhibits IFN-γ-stimulated cell-intrinsic defense against *Cryptosporidium*, an apicomplexan parasite and a leading cause of infectious diarrhea and diarrheal-related death in young children worldwide. Furthermore, antisense oligonucleotides designed to block Prdm1-XR_001779380 interaction can promote the IFN-γ response in murine neonatal IECs, leading to enhanced cell-intrinsic anti-*Cryptosporidium* defense.

**IMPORTANCE:**

Compared with adults, the innate antimicrobial defense of intestinal epithelium in neonates and infants is typically reduced, leading to increased susceptibility to infection; however, the underlying mechanisms remain incompletely understood. *Cryptosporidium* is a leading cause of infectious diarrhea and diarrheal-related death in children worldwide. Prdm1 is a DNA-binding protein that is expressed in neonatal, but not adult, intestinal epithelium. In our previous study, we found that Prdm1 recruits XR_001779380 to form the Prdm1/Stat1/Pias1 complex. Formation of this complex results in the suppression of IFN-γ-stimulated gene transcription in neonatal IECs. In this study, we further investigated the impact of Prdm1 expression on IFN-γ-stimulated cell-intrinsic anti-*Cryptosporidium* defense in neonatal IECs. We also explored the potential of RNA-based therapeutics targeting Prdm1-RNA interactions to enhance cellular response to IFN-γ. Our findings support that antisense oligonucleotides targeting the Prdm1-XR_001779380 interaction promote IFN-γ-stimulated gene transcription and enhance cell-intrinsic defense against *Cryptosporidium* infection.

## INTRODUCTION

Epithelial cells along the gastrointestinal mucosal surface serve as the first line of defense against pathogen infection in both infants and adults (1). Upon pathogen infection, activation of innate immune receptors in intestinal epithelial cells (IECs) triggers the upregulation of antimicrobial factors, the secretion of cytokines and chemokines, and the infiltration of immune cells, which contribute to direct antimicrobial action or the activation of adaptive immune responses. Additionally, IECs are targets of mucosal immune mediators released by immune cells residing in the gastrointestinal mucosa (1, 2). This communication network is essential for maintaining intestinal homeostasis and involves important immune mediators. One such mediator is the family of interferons (IFNs), which includes IFNs of type I (e.g., IFN-α and IFN-β), type II (IFN-γ), and type III (IFN-λ family) (3). IECs can produce various type I and III IFNs and act on themselves in an autocrine manner (4). However, IECs do not produce IFN-γ; IFN-γ released from immune cells in the mucosa plays a pivotal role in intestinal defense against pathogen infection (5–7).

Human neonates and infants have often been described as immunodeficient, but research over the past decade has shown that immune responses in early life cannot simply be categorized as such (8–10). Increasing evidence suggests that the reduced responsiveness may be attributed to multiple factors, including development-associated epigenetic processes, negative regulators, and differences in the development of immune cells in early life relative to adulthood (9–12). The expression of pattern-recognizing receptors and key elements of their associated signaling pathways in the innate immune network appears to be fully developed in IECs during early life (13, 14). Specifically, while Jak-Stat signaling is well-developed, IFN-γ-mediated antimicrobial defense is often reduced in the gastrointestinal tract of infants (14, 15). However, the underlying mechanisms that lead to the suppression of IFN-γ-mediated host defense in neonates and infants have not yet been fully delineated.

Once IFN-γ signaling is activated, complex regulatory mechanisms are initiated to maintain a balance between the intensity and duration of the response, ensuring homeostasis. These regulatory mechanisms involve the activation or repression of transcription, as well as the activation, inhibition, internalization, localization, or degradation of protein mediators and their interactions with other regulatory factors (16). Several of these processes are precisely regulated by long non-coding RNAs (lncRNAs), which are transcripts with limited coding capacity but highly versatile functions (17–19). LncRNAs exert their effects through specific interactions with various cellular factors, including proteins, DNA, and other RNA molecules (20, 21). They can regulate immune responses and inflammation epigenetically by binding to chromatin modifiers or transcription-related factors (10, 21). Some lncRNAs are induced in innate immune cells and are likely to play key roles in regulating innate defense mechanisms (22–25)

*Cryptosporidium*, an apicomplexan parasite that infects the gastrointestinal epithelium and other mucosal surfaces in humans, is a leading cause of infectious diarrhea and diarrheal-related death in children in low- and middle-income countries and a major cause of waterborne disease in the US (26). Human cryptosporidiosis, primarily caused by *C. parvum* and *C. hominis*, is responsible for approximately 50,000 deaths annually, with an estimated global burden of 12.8 million disability-adjusted life years (27–29). Humans become infected with *Cryptosporidium* by ingesting oocysts. After excystation in the gastrointestinal tract, infective sporozoites are released, with each sporozoite attaching to the apical membrane of IECs and forming an intracellular but extracytoplasmic parasitophorous vacuole in which the parasite continues to develop (30). Therefore, cell-intrinsic defense is the first line of mucosal defense against *Cryptosporidium* infection, playing a critical role in the initiation, regulation, and resolution of both innate and adaptive immune reactions (30). All three types of IFN signaling are activated in the intestinal epithelium following *Cryptosporidium* infection (31–33). Each IFN type plays a distinct role in intestinal anti-parasitic defense: type II (IFN-γ) and III IFNs are protective, while type I IFN signaling has a pro-parasitic effect (32–34). The suppression of IFN-γ-mediated antimicrobial defense in early life, compared with adults (8–10), may contribute to the heightened susceptibility of young children to *Cryptosporidium* infection.

We recently observed that murine lncRNA XR_001779380 interacts with Snf5, a component of the Stat1/Swi/Snf5 complex, to promote IFN-γ-stimulated gene transcription in murine IECs (35). Interestingly, PR/SET domain 1 (Prdm1, also known as Blimp1), a DNA-binding protein expressed in neonatal IECs or induced in AIDS patients (36, 37), acts as a tether/sequestration factor by recruiting XR_001779380 to form the Prdm1/Stat1/Pias1 complex. This interaction between XR_001779380 and Prdm1 attenuates IFN-γ-stimulated gene transcription, thereby contributing to the suppression of epithelial cell-intrinsic defense in neonatal intestine against intracellular pathogens, such as *Cryptosporidium* (35). In this study, our data demonstrate that Prdm1 suppresses IFN-γ-mediated, cell-intrinsic antimicrobial defense in neonatal murine IECs. Moreover, antisense oligonucleotides (ASOs) targeting the Prdm1-XR_001779380 interaction can promote IFN-γ-stimulated gene transcription and enhance cell-intrinsic defense against *Cryptosporidium* infection.

## RESULTS

### Knockout of Prdm1 in neonatal IECs results in significant alterations in the gene expression profile induced by IFN-γ

**Fig 1:**
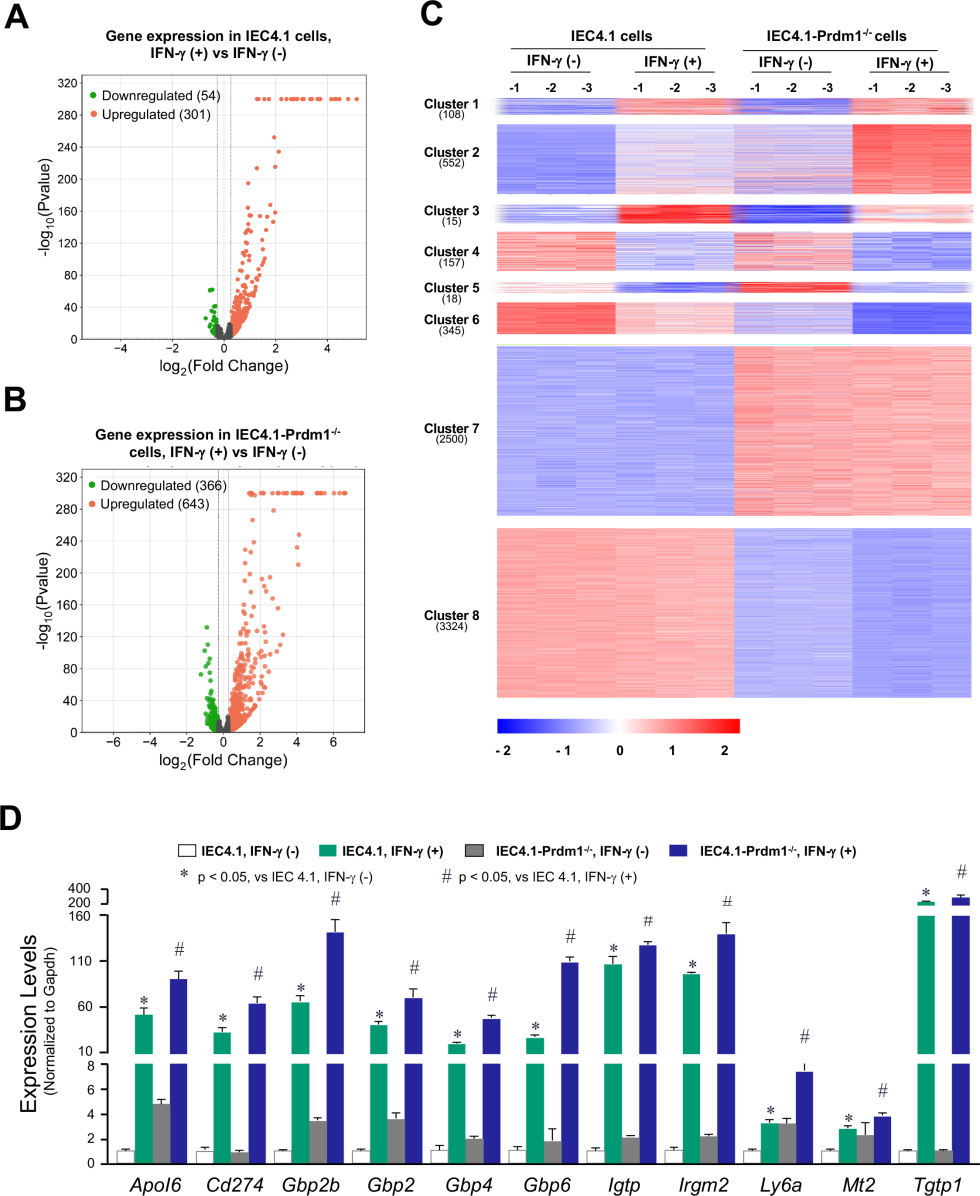
Knockout of PRDM1 in IEC4.1 cells alters gene expression profiles induced by IFN-γ. (**A and B**) Volcano plots depicting the differentially expressed genes in cells following IFN-γ stimulation. Cells were treated with IFN-γ (1 ng/mL) for 2 h followed by RNA-Seq analysis. Volcano plots depicting the differentially expressed genes in IEC4.1 cells (**A**) and IEC4.1-Prdm1^−/−^ cells (**B**) with versus without IFN-γ stimulation. Data are from three biological replicates (3 RNA-seq replicates each group). (**C**) Heatmap representing altered expression levels of selected gene clusters in response to IFN-γ stimulation in IEC4.1 cells vs IEC4.1-Prdm1^−/−^ cells. Genes whose expression levels in response to IFN-γ stimulation were significantly different (*P* < 0.05) in IEC4.1 cells vs IEC4.1-Prdm1^−/−^ cells were selected and clustered. Their expression levels in the non-IFN-γ-treated IEC4.1 cells and IEC4.1-Prdm1^−/−^ cells were also shown. Data are from three RNA-Seq biological replicates for each group. (**D**) The expression levels of these selected defense genes in IEC4.1 cells following IFN-γ stimulation were further validated by qRT-PCR. Data are from three biological replicates and presented as mean values ± SD. *P* values were determined by two-way ANOVA test.

To investigate the role of Prdm1 in regulating IFN-γ-stimulated gene expression in neonatal IECs, we employed IEC4.1 cells for our *in vitro* experiments. IEC4.1 cells are transformed but non-tumorigenic IECs from neonatal mice (5–7 days old) (38) and endogenously express Prdm1 (35). Using the CRISPR/Cas9 system, we previously generated stable IEC4.1 cells deficient in Prdm1 (IEC4.1-Prdm1^−/−^) (35). We cultured IEC4.1 and IEC4.1-Prdm1^−/−^ cells to approximately 90% confluence, stimulated them with IFN-γ (1 ng/mL for 2 h), and then performed RNA-Seq analysis to assess the genome-wide gene expression profiles induced by IFN-γ. Consistent with the results from previous studies (39–41), IFN-γ stimulation significantly altered the gene expression profiles in IEC4.1 cells compared with non-IFN-γ-treated IEC4.1 cells. A total of 301 genes were upregulated (e.g., *Ido1*, *Gbp2*, *Igtp*, *Irgm2*, and *Tgtp1*), and 54 genes were downregulated (e.g., *Bcl6*, *Edn1*, *Artn*, and *Zfp36l1*) (Fig. 1A). In IFN-γ-treated IEC4.1-Prdm1^−/−^ cells, a total of 643 genes were upregulated, and 366 genes were downregulated compared with non-IFN-γ-treated IEC4.1-Prdm1^−/−^ cells (Fig. 1B). A full list of these genes and their expression levels from the RNA-Seq analysis can be found in Table S1, and the whole RNA-seq data set is deposited in the Gene Expression Omnibus (GEO) database repository under the accession number GSE245345.

A significant difference in the gene expression profiles induced by IFN-γ was observed between IEC4.1 and IEC4.1-Prdm1^−/−^ cells. A detailed comparison of these differentially expressed genes revealed eight distinct gene expression patterns (referred to as gene clusters, and the gene list for each cluster is available in Table S2). Gene cluster #1 includes 108 genes, those expression levels were upregulated in IFN-γ-treated IEC4.1 cells compared with control cells (IEC4.1 cells without IFN-γ treatment), and were similarly upregulated in IFN-γ-treated IEC4.1-Prdm1^−/−^ cells at levels comparable to those in IFN-γ-treated IEC4.1 cells. Gene cluster #2 consists of 552 genes that were upregulated in IFN-γ-treated IEC4.1 cells, with even higher upregulation observed in IFN-γ-treated IEC4.1-Prdm1^−/−^ cells compared with IFN-γ-treated IEC4.1 cells. Gene cluster #3 includes 15 genes, and those expression levels were upregulated in IFN-γ-treated IEC4.1 cells but were lower in IFN-γ-treated IEC4.1-Prdm1^−/−^ cells compared with IFN-γ-treated IEC4.1 cells. Gene cluster #4 consists of 157 genes, which were downregulated in IFN-γ-treated IEC4.1 cells at similar levels to those in IFN-γ-treated IEC4.1-Prdm1^−/−^ cells (with no significant difference between the two groups). Gene cluster #5 includes 18 genes, and those expression levels were downregulated in IFN-γ-treated IEC4.1 cells but were significantly higher in IFN-γ-treated IEC4.1-Prdm1^−/−^ cells compared with IFN-γ-treated IEC4.1 cells. Gene cluster #6 includes 345 genes, which were downregulated in IFN-γ-treated IEC4.1 cells and further downregulated in IFN-γ-treated IEC4.1-Prdm1^−/−^ cells compared with IFN-γ-treated IEC4.1 cells. Gene cluster #7 consists of 2,500 genes, whose expression levels did not change in IFN-γ-treated IEC4.1 cells compared with the control cells (IEC4.1 cells without IFN-γ treatment) but were upregulated in IFN-γ-treated IEC4.1-Prdm1^−/−^ cells compared with IFN-γ-treated IEC4.1 cells. Gene cluster #8 includes 3,324 genes, and those expression levels were unchanged in IFN-γ-treated IEC4.1 cells but were downregulated in IFN-γ-treated IEC4.1-Prdm1^−/−^ cells. These gene clusters are visualized in the heatmap (Fig. 1C), and the genes for each cluster are listed in Table S2.

The Go-pathway analysis reveals that the majority of genes with differential expression levels between IFN-γ-treated IEC4.1 cells and IFN-γ-treated IEC4.1-Prdm1^−/−^ cells are involved in the regulation of innate immunity and IFN-γ-stimulated defense responses (Fig. S1A; Table S2). Specifically, genes in cluster #2, which were upregulated in IFN-γ-treated IEC4.1 cells and further upregulated in IFN-γ-treated IEC4.1-Prdm1^−/−^ cells, include many immunity-related genes (*IRGs*) and guanylate binding proteins (*Gbps*). Therefore, we focused most of our subsequent experiments on the genes in cluster #2. The top 16 of these selected genes are shown in the heatmap (Fig. S1B). The expression levels of selected cluster #2 genes in IEC4.1 cells and IEC4.1-Prdm1^−/−^ cells following IFN-γ stimulation were further validated by qRT-PCR (Fig. 1D).

### Overexpression of Prdm1 in IECs on the expression of cluster #2 genes in response to IFN-γ

**Fig 2:**
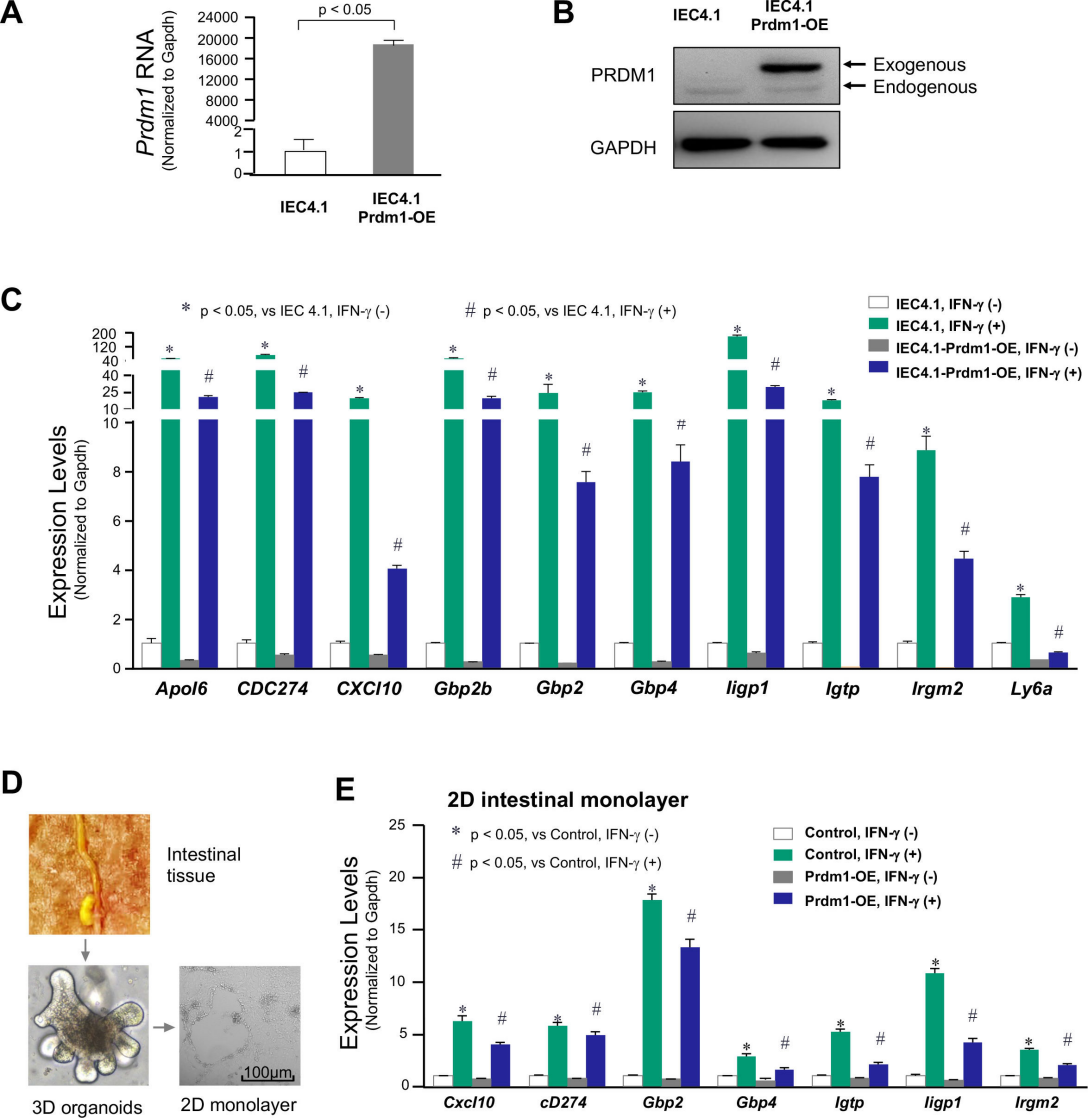
Overexpression of PRDM1 in IECs on the expression of cluster #2 genes induced by IFN-γ. (**A and B**) Overexpression of Prdm1 in IEC4.1 cells. Cells were transfected with the construct expressing Prdm1 for 24 h. Cells transfected with the empty construct were used for control. Forced expression of Prdm1 in IEC4.1 cells was validated by quantitative real-time PCR (qRT-PCR) (**A**) and Western blotting (**B**). (**C**) The expression levels of selected defense genes in cluster #2 in response to IFN-γ stimulation in IEC4.1 cells with forced expression of Prdm1. IEC4.1 cells were transfected with the construct expressing Prdm1 or the empty construct for 24 h, followed by treatment with IFN-γ (1 ng/mL) for 2 h. Genes whose expression levels were significantly altered between IFN-γ-treated IEC4.1-empty and IFN-γ-treated IEC4.1-Prdm1^−/−^ cells were selected, and their expression levels measured by qRT-PCR. Expression levels of Prdm1 in each group were also measured. Data are from three biological replicates and presented as mean values ± SD. *P* values were determined by two-way ANOVA test. (**D and E**) The expression levels of selected defense genes in cluster #2 in response to IFN-γ stimulation in the 2D intestinal monolayers isolated from adult mice with forced expression of Prdm1. Freshly isolated intestinal crypt/villus units from adult mice (6 weeks old) were incubated to form 3D enteroids, which were further developed to intestinal epithelial monolayers. Representative phase images of 3D enteroids and 2D monolayers are shown (**D**). The 2D monolayers were transfected with the construct expressing Prdm1 or the empty construct for 24 h, followed by treatment with IFN-γ (1 ng/mL) for 2 h. The expression levels of selected genes were measured by qRT-PCR (**E**). Data are from three biological replicates and presented as mean values ± SD. *P* values were determined by two-way ANOVA test. Bars = 100 µm.

We generated stable IEC4.1 cells overexpressing Prdm1 (IEC4.1-Prdm1-OE) and examined the gene expression profile induced by IFN-γ. The mouse Prdm1 sequence of 2,571 nt was inserted into the pCMV6-Entry construct for transfection of IEC4.1 cells. Stably transfected cells were selected using antibiotic resistance, and overexpression of Prdm1 was confirmed by qRT-PCR (Fig. 2A) and Western blotting (Fig. 2B). We then stimulated both IEC4.1 cells and IEC4.1-Prdm1-OE cells with IFN-γ (1 ng/mL for 2 h). Using qRT-PCR, we measured the expression levels of selected cluster #2 genes in the treated cells. As expected, we observed an inhibitory effect of Prdm1 overexpression on the expression of these selected genes induced by IFN-γ (Fig. 2C). While induction of these selected genes was detected in IFN-γ-treated IEC4.1 cells, significantly lower expression of these genes was observed in IFN-γ-treated IEC4.1-Prdm1-OE cells (Fig. 2C). Previous studies have shown the absence of Prdm1 in the intestinal epithelium of adult mice (36). We then expressed Prdm1 in cultured 2D intestinal monolayers isolated from adult mice (Fig. 2D). Similar to our findings in IEC4.1 cells, overexpression of Prdm1 in adult 2D intestinal monolayers inhibited the upregulation of these selected cluster #2 genes induced by IFN-γ (Fig. 2E).

### Development of phosphorodiamidate morpholino ASOs (PM-ASOs) to interfere with the Prdm1-XR_001779380 interaction in response to IFN-γ in neonatal IECs

**Fig 3:**
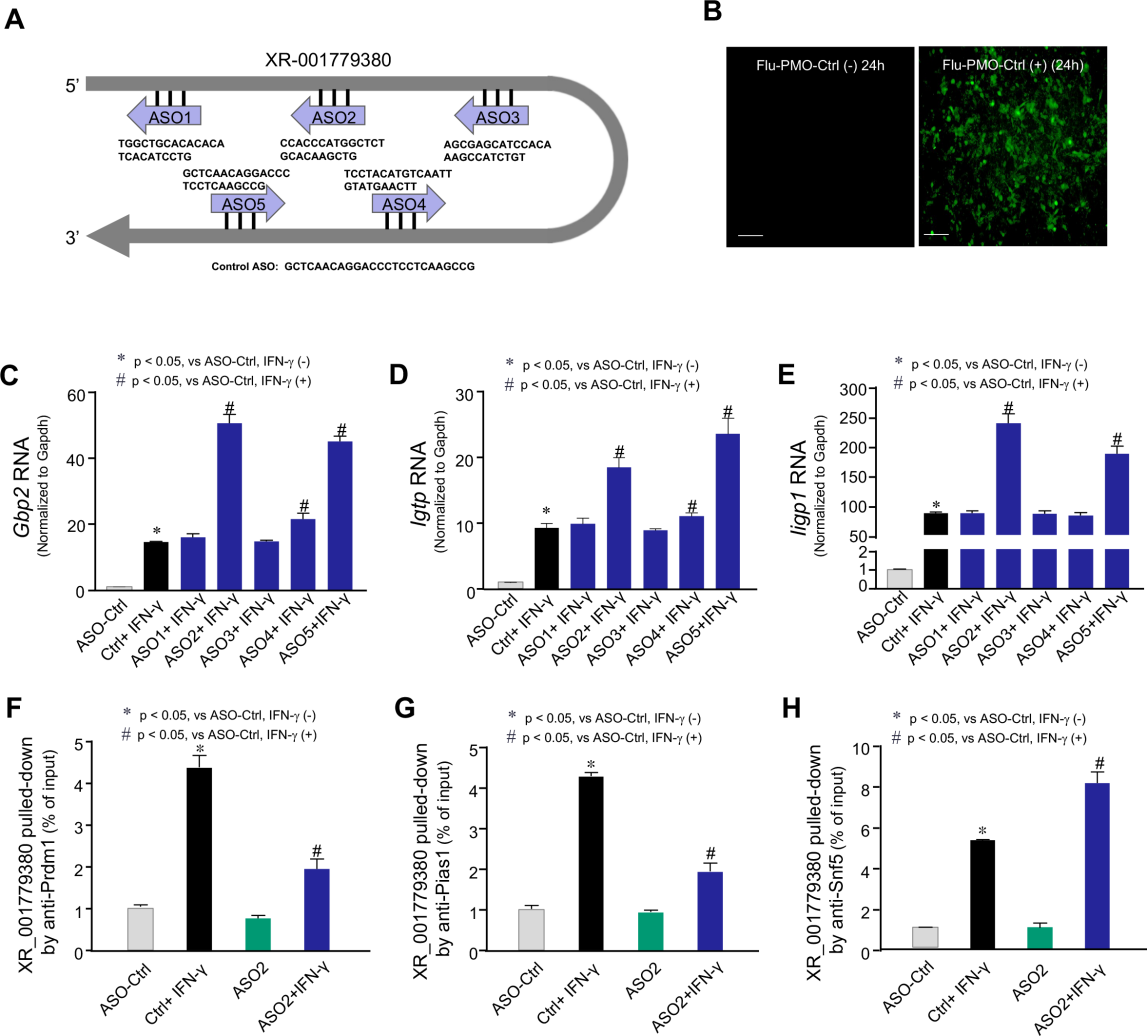
Development of antisense oligonucleotides (ASOs) to interfere with XR_001779380-Prdm1 interaction in IECs induced by IFN-γ. (**A**) *In silico* prediction of the interaction between Prdm1 and XR_001779380. Using the catRAPID online platform, four regions of the XR_001779380 sequence were identified with the potential to interact with Prdm1. (**B**) Delivery of the PM-ASO into IEC4.1 cells. IEC4.1 cells were transfected with the fluorescent-labeled PM-ASO-Ctrl for 24 h. Presence of labeled PM-ASO-Ctrl in cultured IEC4.1 cells as visualized under fluorescent microscopy. A representative image is shown. (**C–E**) Effects of designed ASOs on IFN-γ-stimulated expression of selected genes in IEC4.1 cells. Cells were treated with various ASOs (including the ASO-Ctrl) for 24 h and then exposed to IFN-γ (1 ng/mL) for 2 h. Expression levels of *Gbp2* (**C**), *Igtp* (**D**), and *Iigp1* (**E**) were measured by RT-PCR. Data are from three biological replicates and presented as mean values ± SD. *P* values were determined by two-way ANOVA test. (**F–H**) Effects of designed ASOs on the interaction between XR_001779380 and Prdm1, Pias1, or Snf5 induced by IFN-γ. IEC4.1 cells were treated with various ASOs for 24 h and then exposed to IFN-γ (1 ng/mL) for 2 h. Interactions between XR_001779380 and Prdm1 (**F**), or Pias1 (**G**), or Snf5 (**H**) were measured using the RNA pull-down and RIP assays. Data are from three biological replicates and presented as mean values ± SD. *P* values were determined by two-way ANOVA test.

We previously found that lncRNA XR_001779380 interacts with both Snf5 and Prdm1 (35). Its interaction with Snf5 promotes Stat1/Swi/Snf-associated gene transcription induced by IFN-γ. In contrast, Prdm1 acts as a tether to interact with XR_001779380, recruiting it to form the Prdm1/Stat1/Pias1 complex. This complex suppresses Stat1/Swi/Snf5 complex-associated gene transcription and intestinal defense in murine neonatal IECs in response to IFN-γ (35). ASOs are single-stranded nucleic acids that specifically bind to their cognate RNA target via Watson–Crick base pairing, and several ASO drugs are now FDA approved (42, 43). PM-ASOs feature a backbone of morpholine rings linked by phosphorodiamidate linkages (44). We then asked whether PM-ASOs targeting the XR_001779380-Prdm1 interaction (but not its interaction with Snf5) could promote IFN-γ-stimulated defense in neonatal IECs. We first map the potential interaction regions of XR_001779380 and Prdm1 based on their sequence and structural features using the catRAPID online platform, which is widely used for predicting RNA-protein interactions, and its prediction was effectively validated in previous studies (45, 46). Four high-scoring regions of XR_001779380 were predicted, and based on these predictions, multiple ASOs targeting these regions were synthesized with morpholino modification by GeneTool LLC (Fig. 3A). A non-specific standard control PM-ASO provided by GeneTool LLC was used as the control (PM-ASO-Ctrl). Their sequences and corresponding targeted regions of XR_001779380 were listed in Table S3. To confirm the successful delivery of the PM-ASOs, the PM-ASO-Ctrl was labeled with carboxyfluorescein by GeneTool LLC. Fluorescent microscopy confirmed the delivery of the fluorescent-labeled PM-ASO-Ctrl in cultured IEC4.1 cells (Fig. 3B). We then investigated the potential effects of these ASOs on IFN-γ-stimulated expression of *Gbp2*, *Igtp,* and *Iigp1*, three top representative cluster #2 genes, in IEC4.1 cells. Cells were treated with the ASOs for 24 h and then exposed to IFN-γ (1 ng/mL, for 2 h). We detected enhanced expression of *Gbp2* (Fig. 3C), *Igtp* (Fig. 3D), and *Iigp1* (Fig. 3E) in cells treated with PM-ASO#2 and PM-ASO#5, which target the 176-227 and 435-486 regions of XR_001779380, respectively (Fig. 3A). This enhanced expression of selected cluster #2 genes in response to IFN-γ was not observed in cells transfected with other PM-ASOs or PM-ASO-Ctrl (Fig. 3C through E). Given the most significant effect of PM-ASO#2 on IFN-γ-stimulated gene expression, we selected PM-ASO#2 for further experiments. Using RNA pull-down and RIP assays, we observed that interactions between XR_001779380 and Prdm1 (Fig. 3F), as well as interactions between XR_001779380 and Pias1 (Fig. 3G), in response to IFN-γ were significantly inhibited in IEC4.1 cells transfected with PM-ASO#2. Treatment with PM-ASO#2 also resulted in a significant increase in IFN-γ-induced interactions between Snf5 and XR_001779380 (Fig. 3H), presumably due to the inhibition of XR_001779380-Prdm1/Pias1 interactions by the PM-ASO#2.

### PM-ASOs interfering with the XR_001779380-Prdm1 interaction on IFN-γ-stimulated gene expression in neonatal IECs

**Fig 4:**
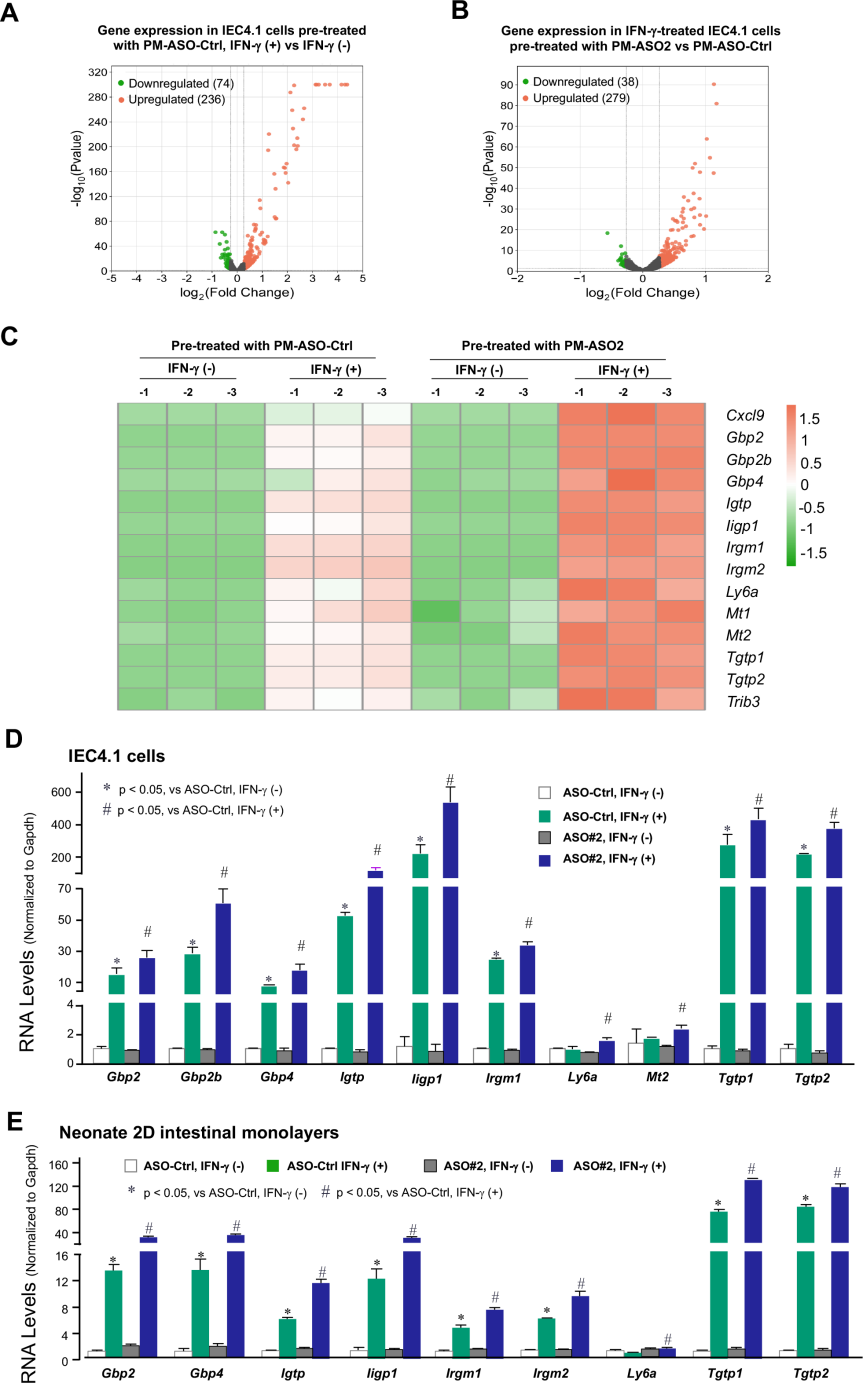
Effects of PM-ASOs interfering XR_001779380-Prdm1 interaction on IFN-γ-stimulated gene expression profiles in IECs. (**A and B**) Effects of PM-ASOs on IFN-γ-stimulated gene expression in IEC4.1 cells. Cells were treated with PM-ASO#2 or the PM-ASO-Ctrl for 24 h and then exposed to IFN-γ (1 ng/mL) for 2 h followed by RNA-Seq. Volcano plots depicting the differentially expressed genes in response to IFN-γ stimulation in IEC4.1 cells treated with PM-ASO-Ctrl (**A**), as well as in IEC4.1 cells treated with PM-ASO#2 vs PM-ASO-Ctrl following IFN-γ stimulation (**B**). Data are from three biological replicates (three RNA-seq replicates each group). Two-tailed Wald tests were performed for statistical analysis. Dashed line indicates a false discovery rate cutoff of 0.05. (**C**) Heatmap representing altered expression levels of selected genes in response to IFN-γ stimulation in IEC4.1 cells treated PM-ASO#2 vs PM-ASO-Ctrl. Genes whose expression levels in response to IFN-γ stimulation were significantly different (*P* < 0.05) in IEC4.1 cells treated with PM-ASO#2 vs PM-ASO-Ctrl were selected. Data are from three RNA-Seq biological replicates for each group. (**D**) Validation of the expression levels of selected defense genes in response to IFN-γ stimulation in IEC4.1 cells treated with PM-ASO#2 vs PM-ASO-Ctrl by RT-PCR. Cells were treated with PM-ASO#2 or the PM-ASO-Ctrl for 24 h and then exposed to IFN-γ (1 ng/mL) for 2 h followed by RT-PCR. Data are from three biological replicates and presented as mean values ± SD. *P* values were determined by two-way ANOVA test. (**E**) The expression levels of these selected defense genes in response to IFN-γ stimulation in the 2D intestinal monolayers isolated from neonatal mice treated with PM-ASO#2 vs PM-ASO-Ctrl. Freshly isolated intestinal crypt units from neonatal mice (6 days old) were cultured to form 3D enteroids, which were further developed to intestinal epithelial monolayers. The 2D monolayers were treated with PM-ASO#2 or PM-ASO-Ctrl for 24 h, followed by treatment with IFN-γ (1 ng/mL) for 2 h. The expression levels of selected genes were measured by qRT-PCR. Data are from three biological replicates and presented as mean values ± SD. *P* values were determined by two-way ANOVA test.

We then investigated the effects of PM-ASO#2 on the genome-wide gene expression profile induced by IFN-γ in IEC4.1 cells. Cells were first transfected with PM-ASO#2 or PM-ASO-Ctrl for 24 h and then cultured for an additional 2 h in the presence or absence of IFN-γ (1 ng/mL), followed by RNA-Seq analysis. Cells pre-treated with PM-ASO-Ctrl or PM-ASO#2 without IFN-γ stimulation showed comparable gene expression profiles (Table S4). Consistent with results of IFN-γ-treated IEC4.1 cells (Fig. 1A), IFN-γ stimulation caused significant alterations in gene expression levels in IEC4.1 cells pre-treated with PM-ASO-Ctrl, including a total of 236 upregulated genes and 74 genes downregulated following IFN-γ stimulation (Fig. 4A). In the absence of IFN-γ, gene expression profiles in IEC4.1 cells treated with PM-ASO-Ctrl and PM-ASO#2 are comparable, suggesting a minimal difference between PM-ASO-Ctrl and PM-ASO#2 treatment on the basal condition (Table S4). However, cells treated with PM-ASO#2 exhibited a distinct gene expression profile in response to IFN-γ. Compared with cells treated with PM-ASO-Ctrl following IFN-γ stimulation, there were 279 genes whose expression levels were further increased, and 38 genes whose expression levels were significantly lower in cells treated with PM-ASO#2 in response to IFN-γ stimulation (Fig. 4B). A full list of these genes is available in Table S4, and the whole RNA-seq data set is deposited in the GEO database repository under the accession number GSE245478. Interestingly, many of the genes whose expression levels were further increased in cells treated with PM-ASO#2 in response to IFN-γ stimulation belong to cluster #2, which was upregulated in IFN-γ-treated IEC4.1-Prdm1^−/−^ cells (Fig. 1C). These selected overlapping genes from cluster #2 are shown in the heatmap (Fig. 4D). The expression levels of these selected cluster #2 genes following IFN-γ stimulation were further validated by qRT-PCR (Fig. 4E). Enhanced expression of these genes in response to IFN-γ was also detected in 2D intestinal monolayers derived from neonatal mice (5 days old) pre-treated with PM-ASO#2 compared with those treated with PM-ASO-Ctrl (Fig. 4F).

### Treatment with PM-ASO#2 does not directly affect the activation of the IFN-γ signal pathway in IECs

**Fig 5:**
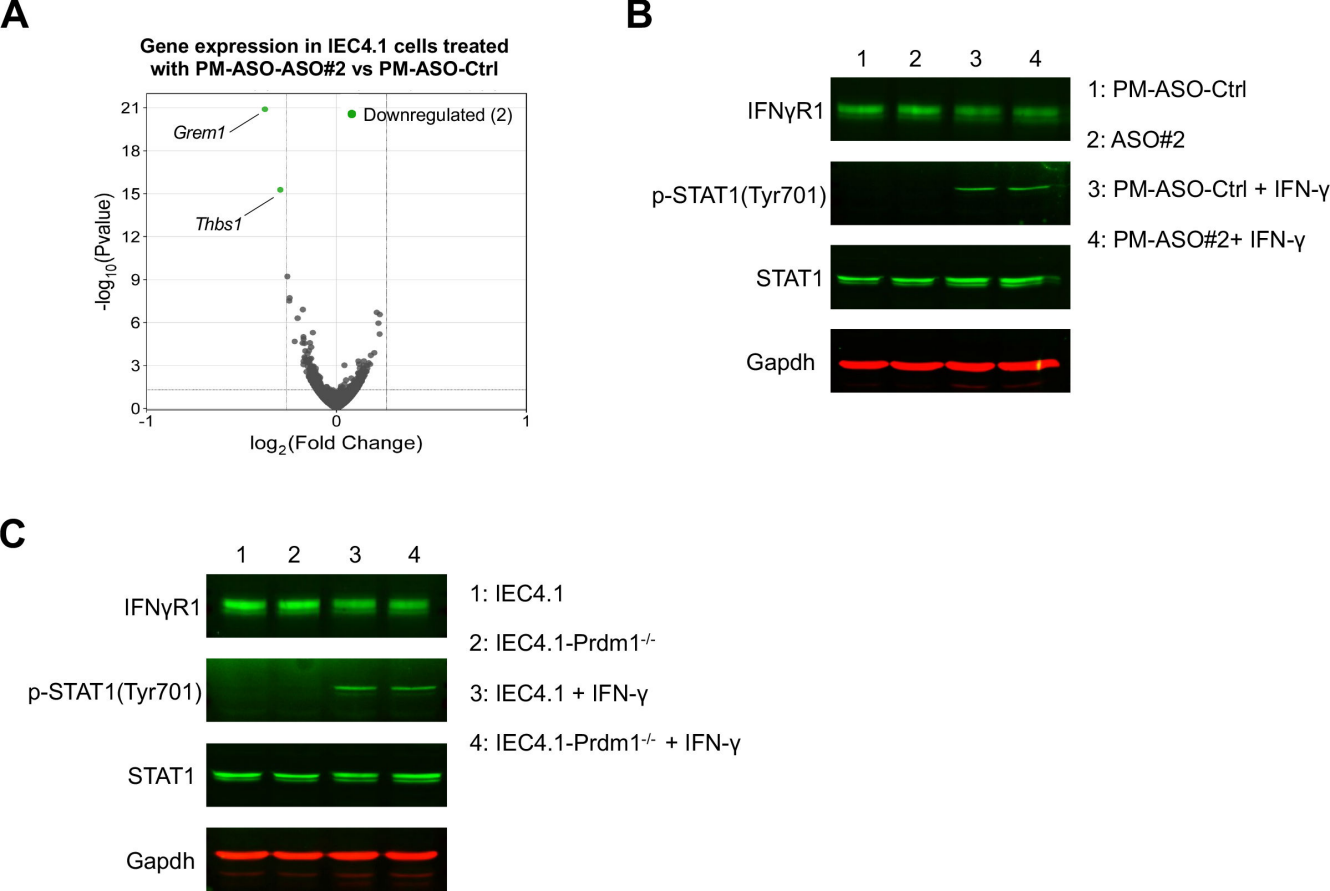
PM-ASOs or Prdm1 knockout do not directly affect the activation of the IFN-γ signal pathway in IECs. (**A**) Gene expression profile in IEC 4.1 cells treated with PM-ASOs. IEC4.1 cells were treated with PM-ASO#2 or PM-ASO-Ctrl for 24 h, followed by RNA-Seq analysis. Volcano plots depicting the expression levels of genes in the cells. Data are from three RNA-Seq biological replicates for each group. (**B and C**) PM-ASO treatment or Prdm1 knockout on the activation of the IFN-γ signal pathway in IEC4.1 cells. For PM-ASO treatment (**B**), IEC4.1 cells were treated with PM-ASO#2 or PM-ASO-Ctrl for 24 h and then exposed to IFN-γ (1 ng/mL) for 2 h. For Prdm1 knockout (**C**), IEC4.1-empty and IFN-γ-treated IEC4.1-Prdm1^−/−^ cells were treated with IFN-γ (1 ng/mL) for 2 h. Protein content of the IFN-γ receptor Ifngr1 and Stat1, as well as p-Stat1 levels, was assessed by Western blot. Gapdh was used as a loading control. Representative gels are shown.

We investigated whether treatment with PM-ASO#2 to interfere with XR_001779380-Prdm1 interaction can directly affect the activation of the IFN-γ signaling pathway in IECs. RNA-Seq analysis revealed no significant changes in gene expression profiles between IEC4.1 cells treated with PM-ASO-Ctrl and PM-ASO#2, including key components associated with the IFN-γ signal pathway, such as downstream JAK/STAT signaling (Fig. 5A). Notably, only two genes (i.e., *Grem1* and *Thbs1*) whose expression levels were lower in the cells treated with PM-ASO#2 compared with the PM-ASO-Ctrl (Fig. 5A). Western blot analysis also showed similar protein levels of the IFN-γ receptor Ifngr1 subunit and IFN-γ-induced Stat1 phosphorylation in IEC4.1 cells treated with PM-ASO-Ctrl and PM-ASO#2, as well as in IEC4.1 and IEC-Prdm1^−/−^ cells IEC4.1 cells (Fig. 5B and C). Therefore, Prdm1 knockout or treatment with PM-ASO#2 does not appear to directly affect the activation of the IFN-γ signal pathway in neonatal IECs.

### Prdm1 knockout or treatment with PM-ASO#2 promotes the recruitment of Stat1 to the promoter regions of associated gene loci in IECs in response to IFN-γ

**Fig 6:**
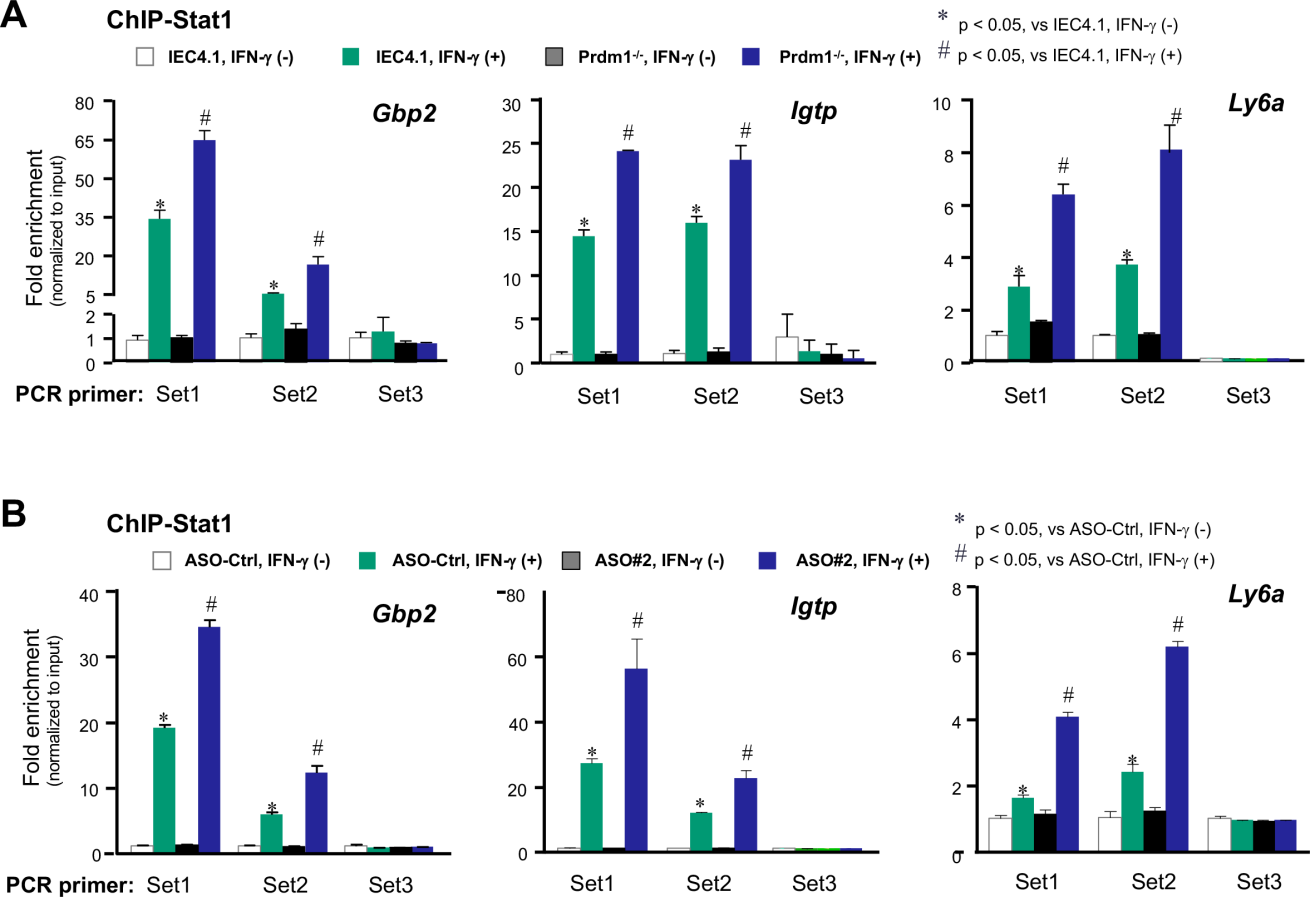
Prdm1 knockout or treatment with PM-ASO#2 promotes recruitment of Stat1 to the promoter regions of associated gene loci in IECs in response to IFN-γ. (**A**) Promoter recruitment of Stat1 induced by IFN-γ stimulation at selected gene loci in IEC4.1 cells and IEC4.1-Prdm1^−/−^ cells. Cells were treated with IFN-γ for 2 h, followed by ChIP analysis using an antibody to Stat1. (**B**) Promoter recruitment of Stat1 induced by IFN-γ stimulation at selected gene loci in IEC4.1 cells treated with PM-ASOs. IEC4.1 cells were treated with PM-ASO#2 or PM-ASO-Ctrl for 24 h and then exposed to IFN-γ stimulation for 2 h, followed by ChIP analysis. Data were from three independent experiments, and statistical significance was determined by two-tailed unpaired Student’s *t*-test.

The canonical IFN-γ signaling utilizes the JAK/STAT pathway to activate STAT1, resulting in the formation of active STAT1 homodimers. These homodimers then bind to gamma-activated sites (GASs) in the promoters (or enhancers) of IFN-γ-stimulated genes (47, 48). We investigated whether Prdm1 knockout or treatment with PM-ASO#2 to interfere with XR_001779380-Prdm1 interaction can promote the recruitment of Stat1 to these gene loci in response to IFN-γ stimulation. We performed chromatin immunoprecipitation (ChIP) assay to measure the recruitment of Stat1 to the promoter regions containing GASs for selected cluster #2 genes (i.e., *Gbp2*, *Igtp*, and *Ly6a*), whose expression levels induced by IFN-γ were further increased in IEC-Prdm1^−/−^ cells or IEC4.1 cells treated with the PM-ASO#2. For each gene locus, we designed two sets of PCR primers (Set1 and Set2) to target the corresponding consensus sequence of GASs within their promoters. Another PCR primer set (Set3) targeting a random region without the GAS sequence was used for control. Consistent with data from previous studies (49), we observed an increase in Stat1 recruitment to the promoter regions containing the GASs for these gene loci in IEC4.1 cells following IFN-γ stimulation (Fig. 6A). The sequences for each PCR primer set to localize the recruitment sites in the promoter regions for each gene (Set1-3) were listed in Table S5. A further increase in Stat1 recruitment was observed in IFN-γ-treated IEC4.1-Prdm1^−/−^ cells (Fig. 6A). Similar results were observed in IEC4.1 cells pre-treated with PM-ASO#2 in response to IFN-γ compared with cells pre-treated with PM-ASO-Ctrl (Fig. 6B). These data suggest that Prdm1 knockout or treatment with PM-ASO#2 promotes the recruitment of Stat1 to the promoter regions of associated gene loci in IECs in response to IFN-γ.

### Prdm1 knockout or treatment with PM-ASO#2 promotes the recruitment of XR_001779380 to the promoter regions of associated gene loci in IECs in response to IFN-γ

**Fig 7:**
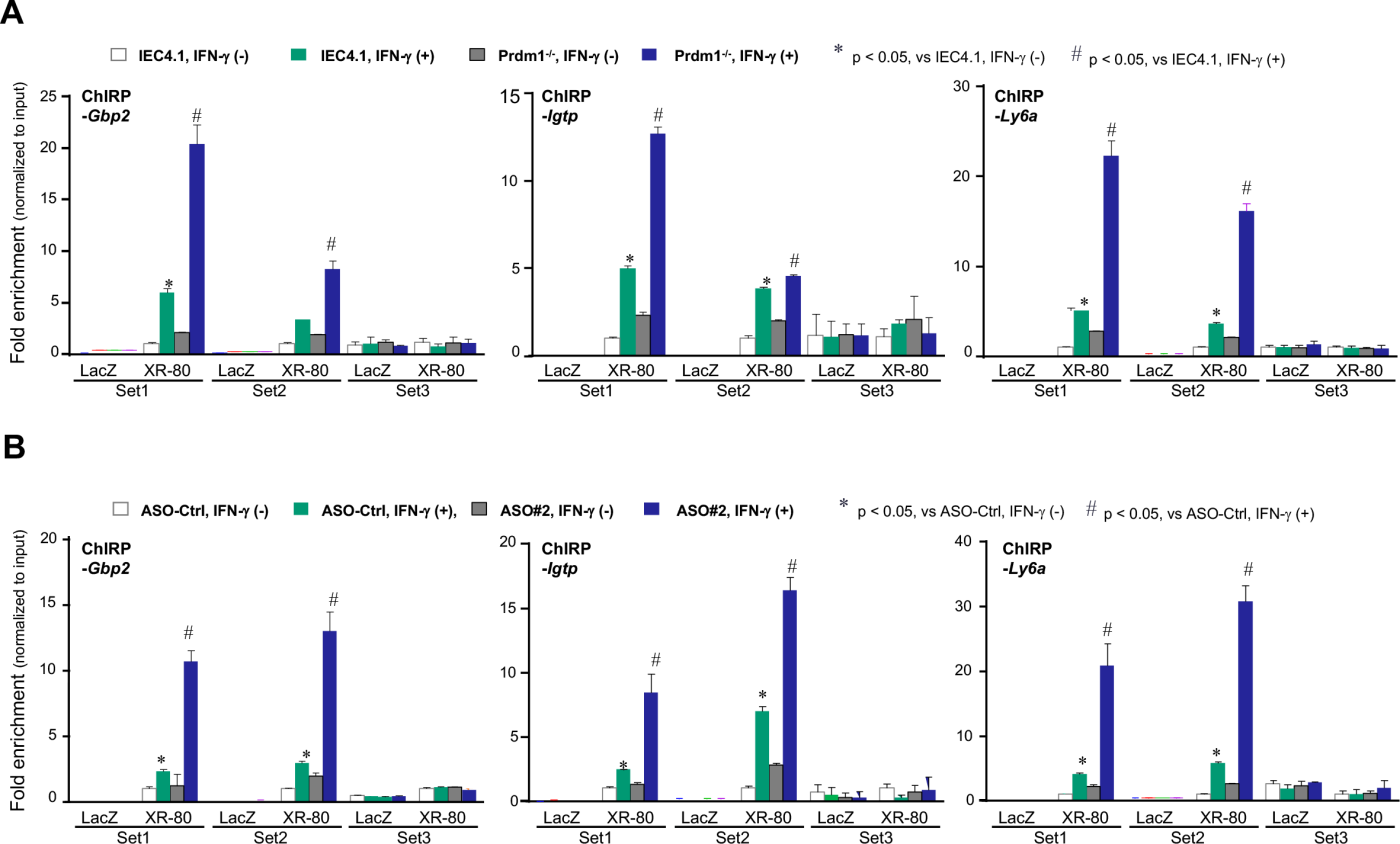
Prdm1 knockout or treatment with PM-ASO#2 promotes recruitment of XR_001779380 to the promoter regions of associated gene loci in IECs in response to IFN-γ. (**A**) Promoter recruitment of XR_001779380 induced by IFN-γ stimulation at selected gene loci in IEC4.1 cells and IEC4.1-Prdm1^−/−^ cells. Cells were treated with IFN-γ for 2 h, followed by ChIRP analysis using probes specific to XR_001779380. (**B**) Promoter recruitment of XR_001779380 induced by IFN-γ stimulation at selected gene loci in IEC4.1 cells treated with PM-ASOs. IEC4.1 cells were treated with PM-ASO#2 or PM-ASO-Ctrl for 24 h and then exposed to IFN-γ stimulation for 2 h, followed by ChIRP analysis. Data were from three independent experiments, and statistical significance was determined by two-tailed unpaired Student’s *t*-test. XR-80 = XR_001779380.

In our previous study, we demonstrated that XR_001779380 interacts with Snf5 to assemble the Stat1/Swi/Snf/XR_001779380 complex, facilitating Stat1/Swi/Snf-associated gene transcription in IECs induced by IFN-γ (35). We then investigated whether Prdm1 knockout or treatment with PM-ASO#2 could promote the recruitment of XR_001779380 to the promoter regions of these gene loci in neonatal IECs in response to IFN-γ stimulation. To address this, we performed ChIRP analysis to measure the recruitment of XR_001779380 to the promoter regions containing gamma-activated sites for selected genes, whose expression levels induced by IFN-γ were further increased in IEC4.1-Prdm1^−/−^ cells or in cells treated with the PM-ASO#2. Biotinylated tiling oligonucleotide probes specific to XR_001779380 were used to purify chromatin fragments, which were then identified via qRT-PCR using the same PCR primers as in the ChIP analysis. As shown in Fig. 7A, increased recruitment of XR_001779380 was observed in the IEC4.1 cells following IFN-γ stimulation. A further increase in XR_001779380 recruitment was observed in IFN-γ-treated IEC4.1-Prdm1^−/−^ cells (Fig. 7A). Similar results were observed in cells pre-treated with PM-ASO#2 in response to IFN-γ compared with cells pre-treated with PM-ASO-Ctrl (Fig. 7B).

### PM-ASOs and manipulation of Prdm1 expression on IFN-γ-mediated intestinal epithelial cell-intrinsic defense against *Cryptosporidium*

**Fig 8:**
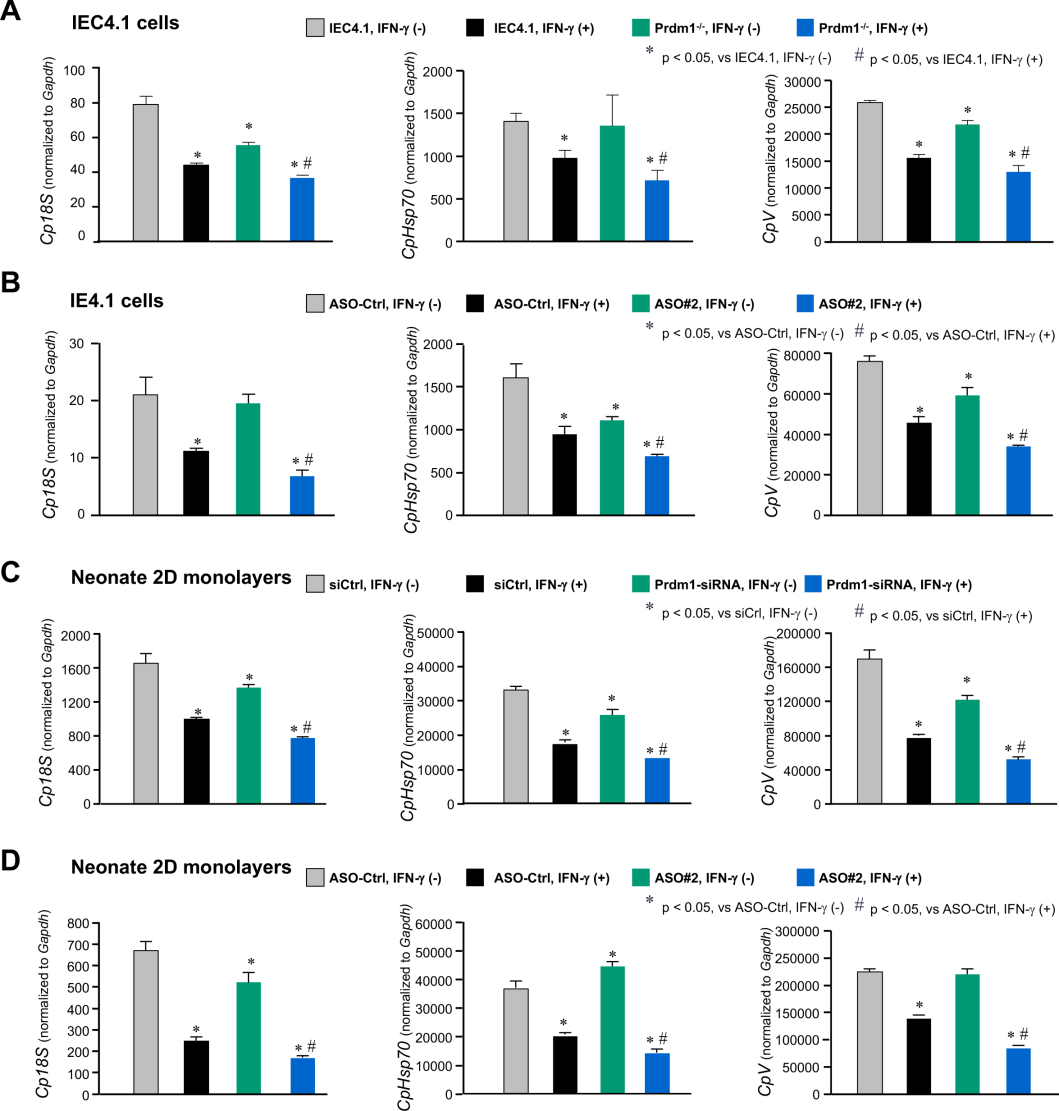
PM-ASOs and manipulation of Prdm1 expression on IFN-γ-mediated intestinal epithelial cell-intrinsic anti-*Cryptosporidium* defense. (**A and B**) Knockout of Prdm1 or MP-ASO treatment on IFN-γ-induced anti-*C*. *parvum* defense. For Prdm1 knockout (**A**), IEC4.1 cells and IEC4.1-Prdm1^−/−^ cells were first treated with IFN-γ (10 ng/mL) for 8 h and then exposed to *C. parvum* for 24 h. For PM-ASO treatment (**B**), IEC4.1 cells were treated with PM-ASO#2 or PM-ASO-Ctrl for 24 h, treated with IFN-γ (10 ng/mL) for 8 h, and then exposed to *C. parvum* for 24 h. Infection burden, reflected by the levels of parasite genes (*Cp18S, CpHsp70,* and *CpV,* normalized to host *Gapdh*)*,* was quantified by RT-qPCR. (**C and D**) Knockdown of Prdm1 or treatment with MP-ASOs on IFN-γ-induced anti-*C*. *parvum* defense in 2D monolayers from neonatal mice. The 2D intestinal monolayers isolated from neonatal mice were treated with siRNAs (Ctrl-siRNA or siRNA_Prdm1 in panel A) or MP-ASOs (PM-ASO#2 or PM-ASO-Ctrl in panel B) for 24 h, treated with IFN-γ (10 ng/mL) for 8 h, and then exposed to *C. parvum* for 24 h. Infection burden was quantified by RT-qPCR. Data in panels A–D are from three biological replicates and presented as mean values ± SD. *P* values were determined by two-way ANOVA test.

Given the importance of Prdm1 expression in regulating IFN-γ-stimulated gene transcription, we investigated whether PM-ASOs targeting the Prdm1-XR_001779380 interaction, as well as manipulation of Prdm1 expression, would impact IFN-γ-mediated cell-intrinsic defense against *Cryptosporidium* in neonatal intestinal epithelium. To assess this, we treated IEC4.1 and IEC4.1-Prdm1^−/−^ cells with IFN-γ for 8 h and then exposed them to *C. parvum* infection for 24 h. Infection burden was quantified by measuring several parasite-origin genes (i.e., *Cp18S*, *CPV*, and *CpHsp70*), normalizing to host *Gapdh* levels via RT-PCR as previously reported (33, 35). Consistent with previous findings, exogenous IFN-γ suppressed *C. parvum* infection in IEC4.1 cells (Fig. 8A). Notably, a significantly lower *C. parvum* infection burden was observed in IFN-γ-pretreated IEC4.1-Prdm1^−/−^ cells compared with IFN-γ-pretreated IEC4.1 cells (Fig. 8A), indicating an enhanced inhibition of infection by IFN-γ in IEC4.1-Prdm1^−/−^ cells. Furthermore, a significantly lower *C. parvum* infection burden was observed in IEC4.1 cells pretreated with IFN-γ and PM-ASO#2, compared to controls (IEC4.1 cells pretreated with IFN-γ and PM-ASO-Ctrl or with IFN-γ alone) (Fig. 8B). Using 2D epithelial monolayers derived from 3D organoids of neonatal mice (5 days old) (as shown in Fig. 2D), we also observed a significant decrease in the infection burden in monolayers treated with IFN-γ (Fig. 8C). A similar reduction in infection burden was seen in monolayers treated with an siRNA that knocked down Prdm1 expression (Fig. 8C). A significant decrease in infection burden was also noted in monolayers pre-treated with IFN-γ and PM-ASO#2 compared with controls (Fig. 8D).

## DISCUSSION

Prdm1 typically functions as a transcriptional repressor to regulate cell differentiation (36) and has been identified as an immunological regulator in various immune cell types, including acting as a critical negative regulator of NK function (50–53). Prdm1 may serve as a master regulator of intestinal epithelium maturation, as it is strongly expressed throughout the epithelium of the embryonic and infant gut, but absent in the adult intestinal epithelium (36). Functionally, Prdm1 orchestrates the extensive reprogramming of the postnatal intestinal epithelium in both mice and humans (36). In this study, our data suggest that Prdm1 expression in the neonatal intestinal epithelium is at least partially associated with a suppressed cellular response to IFN-γ stimulation, which may contribute to the inhibition of IFN-γ-mediated intestinal epithelial cell-intrinsic antimicrobial defense in infants. Accordingly, ASOs targeting Prdm1-mediated recruitment of XR_001779380 can enhance IFN-γ response in neonatal intestinal epithelial cells, thereby promoting cell-intrinsic defense against *Cryptosporidium* (Fig. S2).

Although Prdm1 is a multifunctional protein that modulates gene expression through different mechanisms, its role in regulating the neonatal intestinal epithelial cell response to IFN-γ appears to be associated with its inhibitory effects on IFN-γ-stimulated promoter recruitment of Stat1. In the canonical IFN-γ signaling, the JAK/STAT pathway activates STAT1, leading to the formation of active STAT1 homodimers, which then bind to GASs in the promoters/enhancers of IFN-γ-stimulated genes (47, 48). Increased recruitment of Stat1 to the promoter regions of several IFN-γ-stimulated gene loci was observed in the IEC4.1-Prdm1^−/−^ cells following IFN-γ stimulation compared with IEC4.1 cells. Correspondingly, analysis of differential gene expression profiles in IFN-γ-treated IEC4.1-Prdm1^−/−^ cells revealed a general suppression of IFN-γ-stimulated gene expression compared with IFN-γ-treated IEC4.1 cells. This was associated with enhanced cell-intrinsic anti-*Cryptosporidium* defense induced by IFN-γ in neonatal IECs treated with an siRNA to knock down Prdm1. Similar results were observed in 2D intestinal epithelial monolayers isolated from neonatal mice. Conversely, overexpression of Prdm1 in 2D intestinal epithelial monolayers derived from adult mice impaired the IFN-γ-induced cell-intrinsic anti-*Cryptosporidium* defense.

Interestingly, the lncRNA XR_001779380 plays a role in Prdm1-mediated suppression of the cellular response to IFN-γ in IECs. In our previous report (35), we observed that XR_001779380 interacts with Snf5 and is incorporated into the Stat1/Swi/Snf5 complex, promoting gene transcription regulated by this chromatin complex in response to IFN-γ stimulation in IECs (35). Prdm1 also interacts with Pias1 to modulate Stat1-mediated gene transcription (53). We found that Prdm1 acts as a tether/sequester, interacting with and recruiting XR_001779380 to form the Prdm1/Stat1/Pias1 complex, which suppresses Stat1/Swi/Snf5 complex-associated and host defense in response to IFN-γ (35). Thus, blocking the Prdm1-XR_001779380 interaction should enhance IFN-γ stimulated gene transcription in neonatal IECs, thereby promoting host anti-*Cryptosporidium* defense.

RNA-based therapeutics exploit various types of DNA or RNA molecules, including ASOs, siRNAs, and RNA aptamers (42, 43). ASOs are single-stranded nucleic acids that specifically bind to their cognate RNA target through Watson–Crick base pairing. Given that many lncRNAs have multiple biological functions through their interactions with distinct partners (20–22), the ASO-based approach to targeting specific lncRNA-protein interactions is superior to siRNAs, which typically induce cleavage and degradation of the targeted RNAs (42, 43). Several ASO drugs are now FDA approved (42–44). In this study, our data suggest that PM-ASOs designed to interfere with the Prdm1-XR_001779380 interaction can enhance IFN-γ stimulated gene transcription and subsequent anti-*Cryptosporidium* defense in neonatal IECs. First, delivery of designed PM-ASOs to IECs is efficient, at least under *in vitro* culture conditions, as evidenced by fluorescent microscopy of fluorescent-labeled PM-ASO-Ctrl. Second, some of these designed PM-ASOs effectively interfere with the specific lncRNA-protein interactions. Finally, PM-ASOs targeting the XR_001779380-Prdm1 interaction promoted IFN-γ-stimulated gene transcription in neonatal IECs and enhanced host anti-*Cryptosporidium* defense. Increased recruitment of Stat1 to the promoter regions of several IFN-γ-stimulated gene loci was observed in IEC4.1 cells treated with the effective PM-ASOs targeting the XR_001779380-Prdm1 interaction. Differential gene expression revealed enhanced expression of many IFN-γ-stimulated genes in these treated IEC4.1 cells. Consequently, an enhanced cell-intrinsic anti-*Cryptosporidium* defense induced by IFN-γ was observed in cultured cells treated with the effective PM-ASOs.

Potential non-specific effects are a concern for any RNA-based therapeutics, including PM-ASOs. Although we cannot completely exclude the possibility of non-specific effects of the effective PO-ASOs targeting the XR_001779380-Prdm1 interaction on host cells, it appears that such effects are minimal. The non-specific PM-ASO control reveals no significant effects on IFN-γ-stimulated gene expression in cells. Additionally, the toxic effects of these PM-ASOs in cultured intestinal epithelial cells were minimal. Nevertheless, further investigation is warranted to determine the biological significance of these PM-ASOs using *in vivo* models—for example, assessing whether they can be effectively delivered into intestinal epithelium and enhance mucosal resistance to *Cryptosporidium* infection in neonatal mice. Furthermore, it would be interesting to explore whether PM-ASOs targeting the Prdm1-XR_001779380 interaction can also regulate gene transcription in neonatal IECs in response to type I and III IFN stimulation. Given the critical role of IFN signaling in intestinal innate defense, it is also important to examine whether Prdm1 expression and PM-ASO targeting Prdm1-XR_001779380 interaction can modulate IFN-mediated cell-intrinsic defense in neonatal or infant intestinal epithelial cells in other hosts and against other pathogens.

## MATERIALS AND METHODS

### PM-ASOs

PM-ASOs targeting XR_001779380 to interfere with XR_001779380-Prdm1 interaction were designed and synthesized as previously reported (54–56). Briefly, potential regions of XR_001779380 and Prdm1 for their interaction were analyzed using the catRAPID online platform (45, 46). Based on the analysis, four high-scoring regions of XR_001779380 were selected, and multiple ASOs targeting these regions were designed and synthesized with morpholino modification from GeneTool LLC. To maximize sequence specificity, PM-ASOs were designed to avoid the polymorphic/mutated regions of the genome and exclude oligos targeting four contiguous guanosine residues for possible Hoogsteen base-pair formation. Additional PM-ASOs targeting these regions with a lower potential and a non-specific ASO was used as the control (PM-ASO-Ctrl). To confirm the successful delivery of designed PM-ASOs, the PM-ASO-Ctrl was also labelled with carboxymethy fluorescence by GeneTool LLC for visualization of intracellular delivery under fluorescent microscopy. Sequences for all the ASOs used in this study were listed in Table S5.

### *C. parvum,* cell lines, infection models, and infection assays

*C. parvum* oocysts of the Iowa strain were purchased from a commercial source (Bunch Grass Farm, Deary, ID). The neonatal intestinal epithelial cell line (IEC4.1) was a kind gift from Dr. Pingchang Yang (McMaster University, Hamilton, Canada). Models of intestinal cryptosporidiosis using IEC4.1 cells and 2D intestinal epithelial monolayers were employed as previously described (33, 35). Isolation of intestinal epithelium and development of 2D monolayers were described as previously described (35). Infection was done in culture medium (DMEM-F12 with 100 U/mL penicillin and 100 µg/mL streptomycin) containing viable *C. parvum* oocysts (oocysts with host cells in a 5:1 ratio). Briefly, cells were grown to approximately 90% confluence and then exposed to medium containing oocysts for 4 h. After incubation, cell cultures were washed with medium to remove unbound, non-infective parasites. The cells were subsequently maintained in fresh medium for an additional period, as specified in each experiment (33, 35). Real-time PCR and immunofluorescence microscopy were used to assay *C. parvum* infection as previously reported (33, 35).

### PCR

For quantitative analysis of RNA expression, comparative real-time PCR was performed as previously reported (33, 35) using the SYBR Green PCR Master Mix (Bio-Rad). The sequences for all the primers described above are listed in Table S5.

### siRNAs, plasmids, and CRISPR/Cas9 knockdown

Custom-designed RNA oligos against Prdm1 and one scrambled RNA (as the siRNA-control) were purchased from Santa Cruz Biotechnology. siRNAs were transfected into IEC4.1 cells (or 2D monolayers) with Lipofectamine RNAimax (Invitrogen). The sequences of siRNAs are listed in Table S5. Generation of the stable IEC4.1-Prdm1^−/−^ cells using the CRISPR/Cas9 approach was described in our previous studies (35). Briefly, Prdm1 was knocked out in IEC4.1 cells using the PRDM1/Blimp-1 CRISPR Plasmids (m) and PRDM1/Blimp-1 HDR Plasmid (m) from Santa Cruz Biotechnology (sc-419334 and sc-419334-HDR, respectively), following the manufacturer’s instructions. Stably transfected cells were selected based on puromycin resistance and further validated by quantitative RT-PCR (qRT-PCR) (35). The resulting IEC4.1-Prdm1^−/−^ cells exhibited phenotypic characteristics similar to those parental IEC4.1 cells, including overall morphology and growth rate (35).

### Western blot

Whole cell extracts and blotting were performed using the standard approach as previously described (33, 35). Anti-Prdm1 (Santa Cruz Biotechnology), anti-pStat1 (Cell Signaling), anti-Stat1 (Cell Signaling), anti-Ifngr1 (Invitrogen), and anti-Gapdh (Santa Cruz Biotechnology) were used.

### RIP, ChIP, and ChIRP analyses

The formaldehyde crosslinking RIP was performed as previously described (33). ChIP analysis was performed with a commercially available ChIP Assay Kit (Upstate Biotechnologies) in accordance with the manufacturer’s instructions. Briefly, cells were treated with trypsin, washed, and crosslinked with formaldehyde, followed by quenching with glycine and centrifugation. Nuclei were isolated, lysed, and solubilized by sonication before preclearing with protein A + G beads and immunoprecipitating RNP complexes with antibody-coated beads. Formaldehyde crosslinks were reversed at 65°C, and the presence of RNA was measured by quantitative, strand-specific RT-PCR using the iCycler iQ Real-time detection system (BioRad). The following antibodies were used for ChIP analysis: anti-Stat1 (Cell Signaling), anti-Snf5 (Invitrogen), and anti-Pias1 (Proteintech). For ChIRP analysis, a pool of tiling oligonucleotide probes with affinity specific to XR_001779380 sequence was used, and glutaraldehyde crosslinked for chromatin isolation. The sequences for each probe are listed in Table S5. The DNA sequences of the chromatin immunoprecipitates were confirmed by real-time PCR using the same primer sets covering the gene promoter regions of interest as for ChIP analysis. A pool of scrambled oligo probes and primers for *LacZ* was used as controls.

### Statistical analysis

All values are given as mean + SE. Means of groups were from at least three independent experiments and compared with Student’s *t* test (unpaired) or the ANOVA test when appropriate. *P-*values < 0.05 were considered statistically significant.

## Data Availability

The RNA-seq data used in this study are deposited in the Gene Expression Omnibus (GEO) database repository under the accession numbers GSE245345 and GSE245478.

## SUPPLEMENTAL MATERIAL

Figure S1 mbio.00773-25-s0001.tif Knockout of PRDM1 in IEC4.1 cells alters expression of genes associated with regulation of innate immunity and IFN-γ-mediated defense response.

Figure S2 mbio.00773-25-s0002.tif ASOs targeting the interaction between XR_001779380 and Prdm1 to enhance IFN-γ-mediated defense in murine neonatal IECs.

Table S1 mbio.00773-25-s0003.xlsx Genes with expression levels significantly altered in response to IFN-γ stimulation.

Table S2 mbio.00773-25-s0004.xlsx Clusters of genes with differential expression in response to IFN-γ stimulation.

Table S3 mbio.00773-25-s0005.xlsx Sequences of designed ASOs and their corresponding targeted regions of XR_001779380.

Table S4 mbio.00773-25-s0006.xls Genes with expression levels significantly altered in response to pretreatment with MP-ASOs and then IFN-γ stimulation.

Table S5 mbio.00773-25-s0007.xlsx Primers.

ASM does not own the copyrights to Supplemental Material that may be linked to, or accessed through, an article. The authors have granted ASM a non-exclusive, world-wide license to publish the Supplemental Material files. Please contact the corresponding author directly for reuse.
