# Supplementary figures and images for: Targeting the interaction between long noncoding RNA XR_001779380 and Prdm1 to enhance IFN-γ immunity in murine neonatal intestinal epithelial cells
Source: mBio. 2025 Jun 11;16(7):e00773-25. doi: 10.1128/mbio.00773-25 (PMC12239576; doi:10.1128/mbio.00773-25)

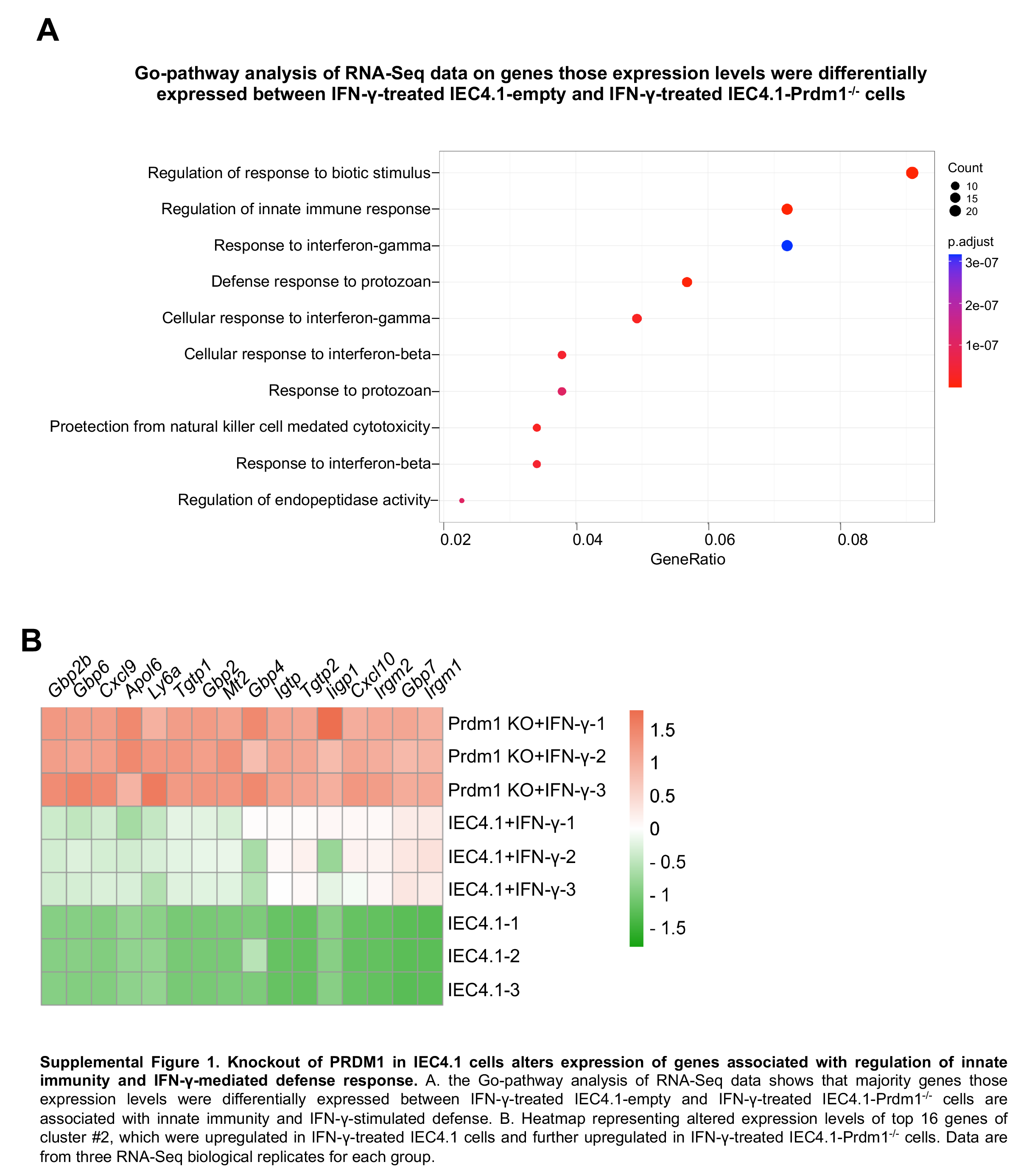

Supplement: Figure S1 — Knockout of PRDM1 in IEC4.1 cells alters expression of genes associated with regulation of innate immunity and IFN-γ-mediated defense response. [file mbio.00773-25-s0001.tif]

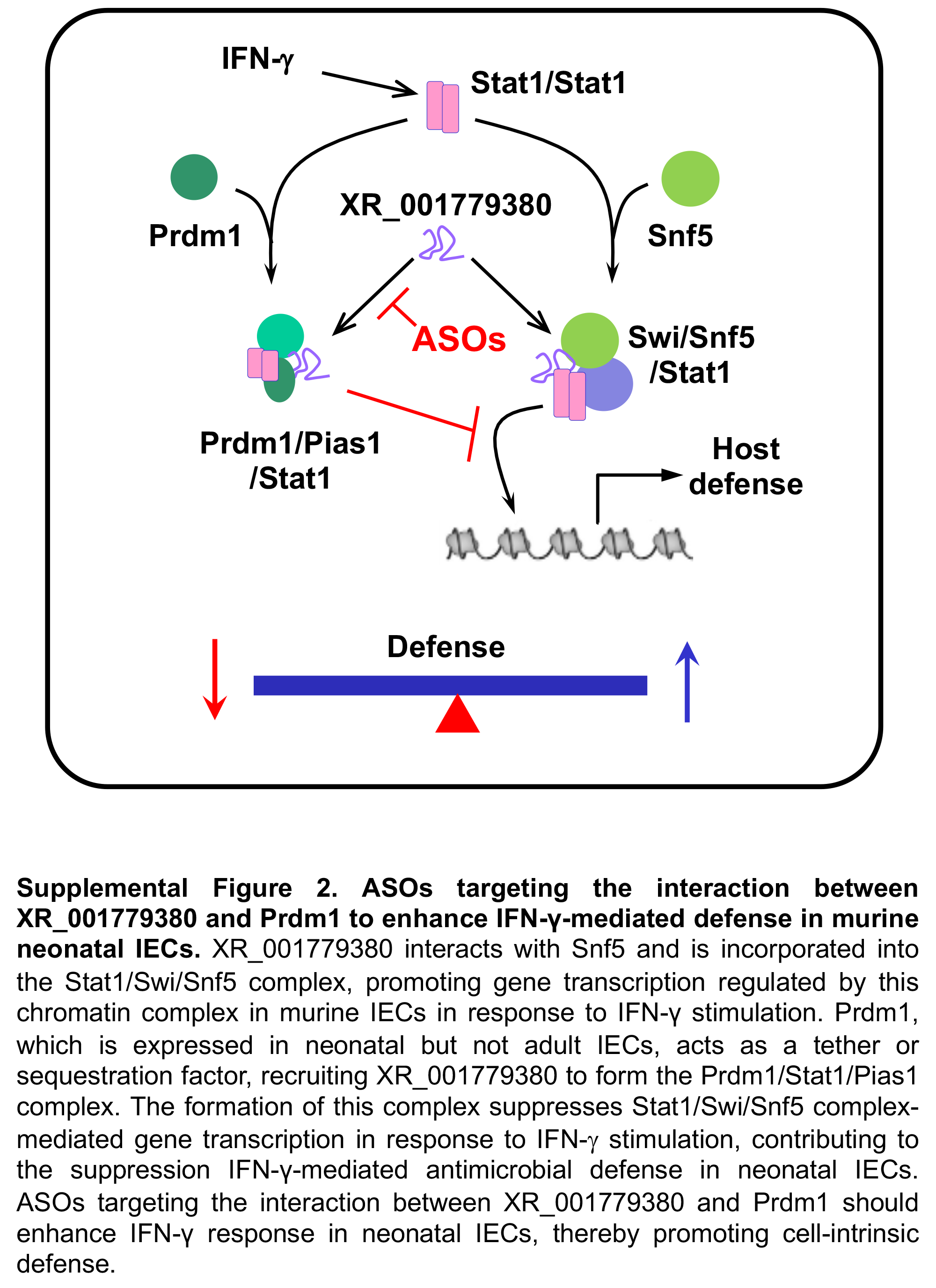

Supplement: Figure S2 — ASOs targeting the interaction between XR_001779380 and Prdm1 to enhance IFN-γ-mediated defense in murine neonatal IECs. [file mbio.00773-25-s0002.tif]
